# Letter to the editor regarding: ‘Association of acute kidney injury stages with in-hospital and long-term mortality in older adults with hip fractures’

**DOI:** 10.1080/07853890.2026.2621467

**Published:** 2026-01-28

**Authors:** Jie Liu, Zhouqi Lao, Qingwei Wang

**Affiliations:** a Department of Orthopedics, Yuyao Traditional Medicine Hospital, Yuyao, Zhejiang, People’s Republic of China; b Xintang Subdistrict Community Health Center, Hangzhou, Zhejiang, People’s Republic of China; c Jiangnan Hospital Affiliated to Zhejiang Chinese Medical University (Hangzhou Xiaoshan Hospital of Traditional Chinese Medicine), Hangzhou, Zhejiang, People’s Republic of China

Dear Editor

We read with great interest the rigorous analysis by Rho et al. [1] demonstrating the graded relationship between AKI stages and mortality in older hip fracture patients. This work compellingly underscores AKI as a critical prognostic indicator in geriatric trauma, highlighting an urgent need for renal-protective strategies. However, to translate these findings into actionable healthcare improvements, we propose three underemphasized dimensions that merit deeper exploration.

## 1. Missed opportunities in preventative care pathways

While the authors rightly stress AKI prevention, their retrospective design inherently overlooks modifiable system-level interventions. Over 35% of patients developed AKI post-admission—a rate exceeding prior reports—suggesting gaps in perioperative fluid management and nephrotoxic medication stewardship. Crucially, no data exists on adherence to evidence-based bundles (e.g. KDIGO guidelines): structured hydration protocols, hemodynamic monitoring, or pharmacist-led medication reviews. A multicentre quality-improvement initiative implementing new fluid balance charts, e-learning tools, and regular AKI alerts with feedback was associated with a 33% relative reduction in hospital-acquired AKI among high-risk trauma patients [2]. Implementing such protocols prospectively could transform the authors’ risk observations into mortality-reducing interventions.

## 2. The sarcopenia–AKI phenotype: unmasking hidden vulnerability

The reliance on creatinine-based AKI staging risks underestimating severity in sarcopenic patients (common in hip fractures), where low muscle mass blunts creatinine rise [3]. This may explain the paradoxically high mortality in stage 1 AKI (HR 1.32–1.64) despite ‘mild’ biochemical impairment. Integrating sarcopenia screening (e.g. calf circumference, SARC-F) would identify a high-risk subgroup needing intensified monitoring. Supporting this, cystatin C and eGFRcys were superior to creatinine‑based measures in predicting long‑term functional decline and frailty progression in older adults, with cystatin C exhibiting substantially stronger associations with deteriorating physical function than creatinine in this population [4]. Beyond validating cystatin C alone, future studies should define sarcopenia-enriched AKI phenotypes integrating dynamic biomarkers. This may optimize rehabilitation allocation and nephrology follow-up intensity.

## 3. Fragmented post-discharge care: the silent mortality driver

The study reports institutionalization in 63.7% of AKI survivors but lacks data on care transitions. Current healthcare systems often fail to fully implement established renal care protocols—including renal function surveillance and medication review—in nursing and primary practice settings [5]. The authors’ observed eGFR decline post-discharge demands coordinated nephrology engagement—yet transfer protocols and specialty access gaps were unstudied. A multidisciplinary co‑management protocol incorporating structured clinical handovers and coordinated specialist consultations has been shown to improve outcomes in older adults with hip fractures, including reductions in mortality and postoperative complications, compared with conventional models of care [6]. Future studies could explore integrated care transition models—such as bundled communication protocols linking orthopedics, primary care, and nephrology—to address survivorship gaps.

Building on Rho et al.’s critical evidence linking AKI severity to mortality, future research should prioritize implementing prospective AKI prevention bundles, developing sarcopenia-cystatin C phenotypes for precise risk stratification, and validating multidisciplinary care transitions. These steps could transform renal injury from a passive outcome to an actionable target, ultimately enhancing survivorship in vulnerable elders with hip fractures.

## Data Availability

Data sharing is not applicable to this article as no data were created or analysed in this study.

## References

[1] Rho JK, Kwon SS, Jang BW, et al. Association of acute kidney injury stages with in-hospital and long-term mortality in older adults with hip fractures. Ann Med. 2026;58(1):2608456. doi: 10.1080/07853890.2025.2608456. 41481400 PMC12777920

[2] Davies A, Srivastava S, Seligman W, et al. Prevention of acute kidney injury through accurate fluid balance monitoring. BMJ Open Qual. 2017;6(2):e000006. doi: 10.1136/bmjoq-2017-000006. PMC5717957 29435501

[3] Koh SY, Jun JH, Kim JE, et al. Sarcopenia, a risk predictor of postoperative acute kidney injury in elderly patients after hip fracture surgery: a retrospective analysis. Medicina (Kaunas). 2024;60(5):745. Published 2024 Apr 29. doi: 10.3390/medicina60050745. 38792928 PMC11122835

[4] Li C, Ma Y, Yang C, et al. Association of cystatin c kidney function measures with long-term deficit–accumulation frailty trajectories and physical function decline. JAMA Netw Open. 2022;5(9):e2234208. doi: 10.1001/jamanetworkopen.2022.34208. 36178684 PMC9526088

[5] May HP, Herges JR, Anderson BK, et al. Posthospital multidisciplinary care for AKI survivors: a feasibility pilot. Kidney Med. 2023;5(12):100734. Published 2023 Oct 5. doi: 10.1016/j.xkme.2023.100734. 37964784 PMC10641567

[6] VanTienderen RJ, Bockelman K, Khalifa R, et al. Implementation of a multidisciplinary ‘code hip’ protocol is associated with decreased time to surgery and improved patient outcomes. Geriatr Orthop Surg Rehabil. 2021;12:21514593211004904. doi: 10.1177/21514593211004904. 35186421 PMC8848070

